# Reduction of pl-CSA through ChSy-2 knockout inhibits tumorigenesis and metastasis of choriocarcinoma in JEG3 cells

**DOI:** 10.7150/ijms.51900

**Published:** 2021-01-01

**Authors:** Juzuo Zhang, Zhilong Chen, Baobei Wang, Jie Chen, Tianxia Xiao, Jian V. Zhang, Shiling Chen, Xiujun Fan

**Affiliations:** 1 College of Biological and Food Engineering, Huaihua University, Huaihua, Hunan 418000, China.; 2 Center for Reproduction and Health Development, Shenzhen Institutes of Advanced Technology, Chinese Academy of Science, Shenzhen, Guangdong 518055, China.; 3 College of Veterinary Medicine, Hunan Agricultural University, Changsha, Hunan.; 4 Department of Gynecology and Obstetrics, Southern Medical University, Guangzhou, Guangdong 510515, China.

**Keywords:** choriocarcinoma, ChSy-2/pl-CSA, tumorigenesis, molecular therapy

## Abstract

**Background:** Placental-like chondroitin sulfate A (pl-CSA) is exclusively expressed in cancerous and placental tissues and is highly correlated with the degree of malignancy. However, the mechanism through which pl-CSA regulates tumorigenesis and metastasis in choriocarcinoma remains unclear.

**Methods:** Stable transfectants of the JEG3 choriocarcinoma cell line, including a negative control (NC) line and a cell line with knockout of the biosynthetic enzyme CS synthase-2 (ChSy-2) (ChSy-2^-/-^), were obtained using CRISPR/Cas9 systems and identified by immunofluorescence, flow cytometry, western blots and enzyme-linked immunosorbent assays (ELISAs). The proliferation, migration, invasion and colony formation of the cells were determined by a cell counting kit, scratch-wound assays, transwell assays and soft agar colony formation assays *in vitro*, respectively. The tumorigenesis and metastasis of choriocarcinoma were also investigated through two xenograft models *in vivo*.

**Results:** The ChSy-2 protein in the ChSy-2^-/-^group was below the detection threshold, which was accompanied a significant reduction in the pl-CSA level. Reducing pl-CSA through ChSy-2 knockout significantly inhibited cell proliferation, migration, invasion and colony formation *in vitro* and tumorigenesis and metastasis of choriocarcinoma, with deceases in tumor volume and metastatic foci and a high percent survival compared to the NC* in vivo*.

**Conclusion:** pl-CSA, as a necessary component of JEG-3 cells, was efficiently reduced through ChSy-2 knockout, which significantly inhibited the tumorigenesis and metastasis of choriocarcinoma. ChSy-2/pl-CSA could be alternative targets for tumor therapy.

## Introduction

Cancer is a serious public health problem worldwide, and choriocarcinoma originating from the trophoblastic tissue in gestation or without pregnancy severely endangers females and even males. The incidence of choriocarcinoma varies in different regions, ranging from 1 in 15,000-50,000 pregnancies in Europe and North America to 1 in 500-20,000 pregnancies in Southeast Asia 1, and is >5 in 10,000 pregnancies in China 2. Although choriocarcinoma is relatively rare, case reports have described it in females and males or from carcinoma *in situ* to metastasis 3. Patients with choriocarcinoma range from 17 to 67 years old, and the asymptomatic period varies from 4 weeks to 25 years 4. Although the prognosis of choriocarcinoma is relatively good, its effects, including damage to multiple organs or injuries and pain accompanying chemotherapy, are harmful and approximately 40% of pregnancies with choriocarcinoma result in neonatal mortality 5. Choriocarcinoma, a highly malignant epithelial cell tumor 6, is mainly characterized by immortalized trophoblast cells with uncontrolled proliferation, migration and invasion and distant metastasis to the blood vessels, lung, liver or brain 7-10. Currently, the molecules involved in the tumorigenesis and metastasis of trophoblast cells are unclear, which is a major challenge to diagnosis and treatment. Due to the lack of reliable target biomarkers, the main therapeutic approach to choriocarcinoma is chemotherapy based on pathological examination and human chorionic gonadotropin (hCG) levels 11 rather than surgery or immunotherapy. Therefore, the degree of malignancy and various clinical phenotypes of choriocarcinoma need to be investigated, and identification of target genes related to occurrence and development, clinical diagnosis and molecular therapy is urgently needed.

Placental-like chondroitin sulfate A (pl-CSA) is a distinct and low-sulfated subtype of chondroitin sulfate (CS), a glycosaminoglycan (GAG) that plays a vital role in tumorigenesis. This receptor can specifically bind with VAR2CSA of *Plasmodium falciparum*-infected erythrocytes or various other ligands and has been associated with the degree of malignancy 12,13. With its typical GlcA-GalNAc (4S) disaccharide unit and relatively low variation, pl-CSA is synthesized in multiple types of cancer 13,14 by chondroitin sulfate synthase (ChSy, especially ChSy-2) and is covalently linked to its core protein 15. As the biosynthetic enzyme of pl-CSA, ChSy-2 can induce high expression of CSA in placental and tumor tissues and may be a key glycosyltransferase 16-18. Targeting ChSy-2 may be an important method to study pl-CSA biofunction because there is no efficient way to directly study glycobiology. Thus, ChSy-2/pl-CSA may be promising cancer biomarkers and play an important role in tumorigenesis and metastasis.

To investigate the role of ChSy-2/pl-CSA in choriocarcinoma, we decreased the pl-CSA content in JEG3 choriocarcinoma cells by deleting the biosynthetic enzyme ChSy-2. The modified JEG3 cells were identified through immunofluorescence, flow cytometry, western blot and enzyme-linked immunosorbent assays (ELISAs). The properties of modified JEG3 cells, including proliferation, migration, invasion and colony formation in soft agar, were investigated through Cell Counting Kit 8 (CCK-8), wound-healing, transwell, and soft agar colony assays, respectively. The roles of ChSy/pl-CSA in the tumorigenesis and metastasis of choriocarcinoma were also investigated using two xenograft mouse models *in vivo*.

## Materials and methods

### Cell culture

The human choriocarcinoma cell line JEG3 was obtained from the American Type Tissue Collection (ATCC number: HTB-36TM, Manassas, VA) and was authenticated using short tandem repeat (STR) analysis for mycoplasma contamination. The cells were routinely cultured in DMEM/F12 (cat. no. 11320033; Thermo Fisher Scientific, Inc., Waltham, MA, USA) supplemented with 10% fetal bovine serum (cat. no. 16000044; Thermo Fisher Scientific, Inc.), penicillin (100 U/ml) and streptomycin (100 μg/ml) (cat. no. 15140122; Thermo Fisher Scientific, Inc.) and maintained in a humidified 5% CO_2_ atmosphere at 37 °C.

### Stable transfection

Stable transfectants were obtained from the JEG3 cell line, including a negative control line (NC) using an empty lentivirus CRISPR/Cas v2 vector with the puromycin resistance gene and a ChSy-2 knockout line (ChSy-2^-/-^) using a plasmid inserting a gRNA of GCCGCGCGGCAACACCAACG (according to online resources at the Zhang Lab website: https://zlab.bio/guide-design-resources) into a vector for targeting exon 1 of ChSy-2 in the coding sequence (AB175496.1).

Plasmid transfection was performed with Lipofectamine® 3000 Reagent (cat. no. L3000008; Thermo Fisher Scientific, Inc.) according to the manufacturer's instructions when the cultured cells reached approximately 70% confluence. The transfected JEG3 cells were screened in medium containing 4 μg/ml puromycin (cat. no. anti-Pr-1, InvivoGen) for 1 week. The cells were isolated, amplified and maintained in 2 μg/ml puromycin for subsequent experiments.

### Preparation of rVAR2

For analysis of pl-CSA, a recombinant VAR2CSA labeled with a 6×His tag (rVAR2), specifically interaction with pl-CSA, was expressed by inserting this fragment into a pET28a (+) vector that was transformed into *Escherichia coli* BL21, and was purified using a Ni^2+^ affinity column (cat. no. DP101-01; Transgen Biotech Co., Ltd., Beijing, China) from the supernatant of bacterial culture. The purified rVAR2 was used in the following experiments.

### Immunofluorescence and flow cytometry

The cultured cells were subjected to immunofluorescence and flow cytometry. Briefly, after fixation, permeabilization and blockage, the cells were successively incubated with a mixture of ChSy-2 rabbit primary antibody (dilution, 1:500; cat. no. ab75052, Abcam) and rVAR2-6×His recombinant protein (dilution, 1:200; Figure 1), and a mixture of FITC-labeled goat anti-rabbit (dilution, 1:1000; cat. no. ab6717; Abcam) and DyLight® 650-labeled anti-6×His tag secondary antibodies (dilution, 1:1000; cat. no. ab117504, Abcam), and followed by counterstain with 0.4 µg/ml DAPI (cat. no. AR1177, Wuhan Boster Biological Technology, Ltd., Wuhan, China). Stained cells were observed and analyzed under a fluorescence microscope (BX53; Olympus Corporation, Tokyo, Japan) using Image-Pro Plus software version 6.0 (Media Cybernetics, Inc., Rockville, MD, USA), and were also analyzed by flow cytometry (CytoFLEX S; Beckman Coulter, Inc., Brea, CA, USA) using FlowJo software (FlowJo 7.6.1; Treestar, Inc., Ashland, OR, USA).

### Proliferative analysis

Cell proliferation was detected using CCK-8 assays 19. Briefly, the cells were seeded into 96-well plates (100 µl/well; 2,500 cells/well) and were cultured at 37 °C in an environment with 5% CO_2_. At 12, 24, 36, 48, 60, 72 and 84 h, 10 µl of CCK-8 solution (cat. no. CK04; Dojindo Molecular Technologies, Inc., Kumamoto, Japan) was added to each well, followed by incubation for 3 h, and the solution color was monitored. When the difference in the color was significant between groups, absorbance was measured at 450 nm.

### Cell migration assay

Cell migration was detected using a scratch-wound assay 20. Briefly, the 90% confluent cell layer was scratched using a 10-µl pipette tip, and detached cells were removed by washing with PBS. The cells were cultured in medium with 2% FBS for 48 h. Images of the scratch wounds were obtained using an inverted phase-contrast microscope (BX53, Olympus Corporation, Tokyo, Japan) equipped with a digital camera at 0, 24 and 48 h. The wound width was determined using Image-Pro Plus software version 6.0.

### Cell invasion assay

Cell invasion was detected using a transwell assay 21 with a Corning® BioCoat™ Matrigel® Invasion Chamber (cat. no., 354480; Corning, Inc., NY, USA). Briefly, the cells were seeded into the transwell upper chamber at a density of 5×10^4^ cells/well in 200 μl of serum-free medium; 600 μl of medium with 10% FBS was added to the lower chambers. Following incubation for 24 h, the noninvasive cells on the upper membrane of the insert were removed using a cotton swab. The migrated cells adhered to the lower membrane surface were fixed and stained with crystal violet dye, and the number of cells was then counted under a microscope in 5 random optical fields.

### Soft agar colony formation assay

The soft agar colony formation assay was used to determine carcinogenesis and malignant transformation *in vitro* 22. Briefly, the cells (10^5^ cells/well) were mixed with 0.3% agar dissolved in growth medium and plated on top of a bottom layer of 0.7% agar dissolved in growth medium in a 6-well plate. A layer of complete medium (~200 μl) was maintained above the upper agar layer and changed every 3 days. After 26 days, the colonies were dyed with crystal violet dye (0.01% solution). Images of the clones were observed using an inverted phase-contrast microscope (BX53, Olympus Corporation, Tokyo, Japan) equipped with a digital camera. The number of clones was determined using Image-Pro Plus software version 6.0.

### Tumorigenesis and metastasis of modified cells *in vivo*

Cell tumorigenesis and metastasis were detected using *in vivo* imaging systems (IVIS, Xenogen Corporation, CA, USA) 23. The cells were engineered to constitutively express the firefly luciferase reporter gene (Fluc2). Then, 1x10^6^ Fluc2-carrying JEG3 cells (JEG3-Fluc2) in 100 μl of PBS were injected into specific-pathogen-free (SPF) 4-6-week-old female nude mice (BALB/c background) either subcutaneously for tumorigenesis or through the tail vein for metastasis. For determination of tumor growth conditions, mice carrying JEG3-Fluc2 cells were intraperitoneally administered 150 mg/kg body weight D-luciferin (cat. no., 103404-75-7; Gold Biotechnology, Inc., St Louis, MO, USA), and the cells emitted a visual light signal that was monitored using IVIS Spectrum 2 days after cell administration and then again 4 days later. The photon flux from the mouse was proportional to the number of light-emitting cells, and the signal was measured to monitor tumorigenesis and metastasis. The region of interest (ROI) of fluorescence imaging was analyzed with an IVIS imaging system. Tumor volume was measured as follows: tumor volume = (tumor length) x (tumor width)^2^/2. The humane endpoint was based on the maximum acceptable total burden of animal models, no longer than 60 days. Animals of all groups in the tumorigenesis experiment were euthanized when the measured volume of subcutaneous tumors was over 1 cm^3^ in any group. Animals in all groups in the metastasis experiment were euthanized on the same day when metastasis was observed in one or two organs in any group. Moreover, the health of the mice was monitored three times a week, with no obvious pain or distress 24.

### Serum hCG measurement

Blood samples were collected from mice administered JEG3 cells through the orbital venous system after anesthesia with isoflurane. The serum was separated from blood samples by centrifugation. Serum hCG levels were determined by radioimmunoassays using an XH6080 radioimmunity analyzer, carried out by Beijing North Institute of Biotechnology Co., Ltd. (Beijing, China).

### ELISA detection of pl-CSA

The pl-CSA level in JEG3 cells was detected as described previously 14. Briefly, approximately 10^6^ well-cultured cells/ml were collected and lysed in lysis buffer for pl-CSA detection. The lysate was added to 96-microwell plates precoated with pl-CSA-BP capture protein, successively followed by incubation with anti-pl-CSA antibody, horseradish peroxidase (HRP)-labeled secondary antibody, and substrate solution [3,3',5,5'-tetramethylbenzidine (TMB)]. After the reaction was stopped with H_2_SO_4_, the absorbance was recorded at 450 nm (OD450 nm) to calculate the pl-CSA content.

### Protein extraction and western blot analysis

Protein extracts were prepared from cells using M-PER™ Mammalian Protein Extraction Reagent (cat. no. 78503; Thermo Fisher Scientific, Inc.) supplemented with EDTA-free complete protease inhibitor cocktail (cat. No. 88265; Thermo Fisher Scientific, Inc.). Protein content was determined using a Micro BCA™ Protein Assay Kit (cat. no. 23235; Thermo Fisher Scientific, Inc.). Protein samples (30 μg) were separated on a 12% SDS-PAGE gel and electrophoretically transferred to PVDF membranes (cat. no. 162-017; Bio-Rad Laboratories, Inc., Hercules, CA, USA). After blockage, the membranes were successively incubated with anti-human ChSy-2 or β-actin rabbit antibodies (dilution, 1:1000, ab75052 or ab16039, respectively; Abcam) and goat anti-rabbit IgG antibody conjugated to horseradish peroxidase (dilution, 1:5000; cat. no. ab97051; Abcam). Protein bands were visualized by enhanced chemiluminescence using SuperSignal™ West Pico Chemiluminescent Substrate (Thermo Fisher Scientific, Inc.) on a ChemiDoc™ XRS+ system (Bio-Rad Laboratories, Inc.). Target blots were normalized to β-actin and semiquantified by densitometry using ImageJ software (v2.1.4.7; National Institutes of Health, Bethesda, MD, USA).

### Statistical analysis

Data are presented as the mean ± standard deviation of at least three independent experiments. The variance between all groups was assessed by ANOVA, and the statistical significance of the differences between groups was assessed using Tukey's multiple comparisons test. Statistical analysis was performed using SPSS software version 19.0 (IBM Corp., Armonk, NY, USA). ** *P*<0.01 and * *P*<0.05 were considered significant.

## Results

### ChSy-2 knockout significantly reduces the pl-CSA content in JEG3 choriocarcinoma cells

**Figure 1:**
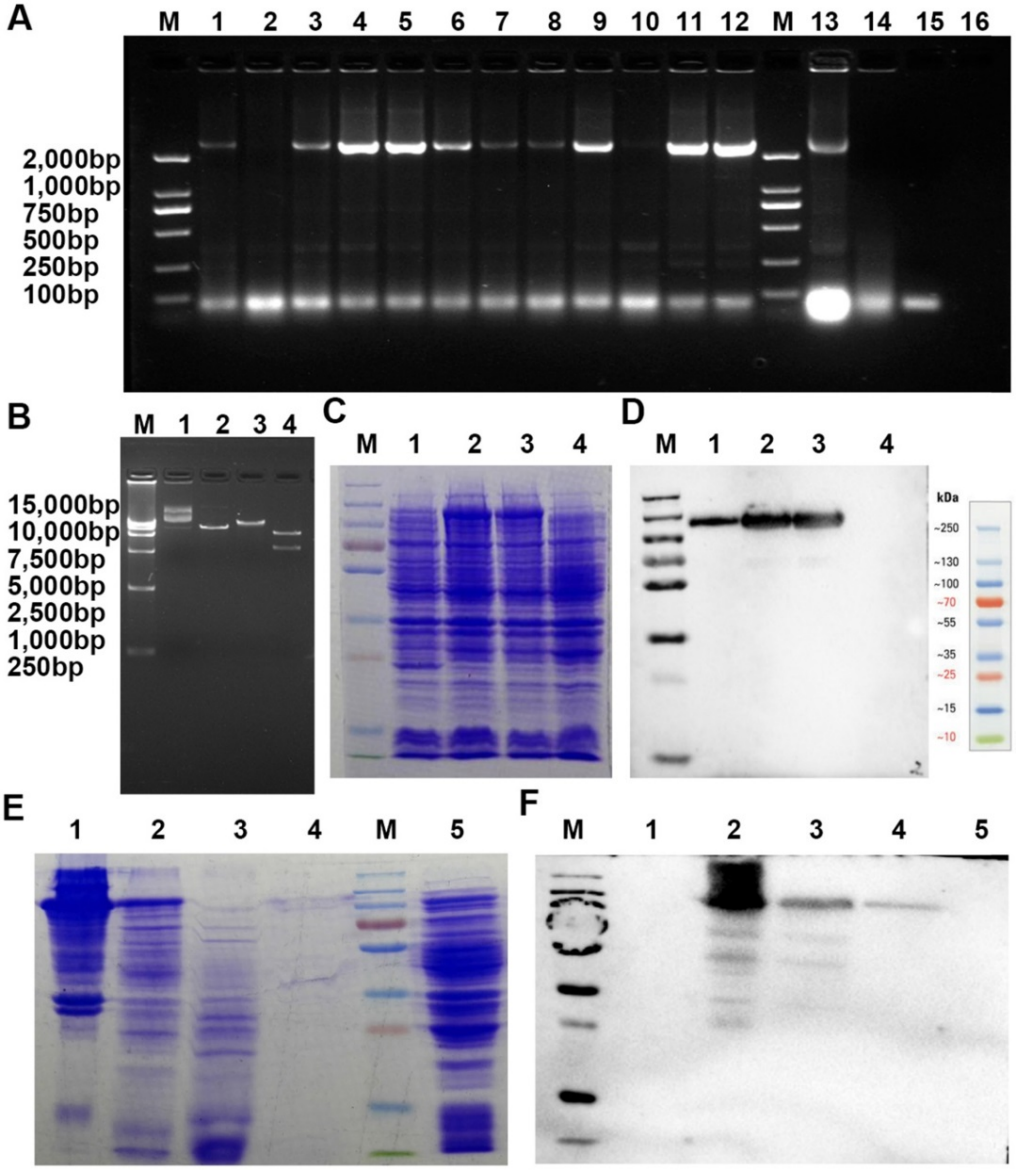
** A recombinant VAR2CSA labeled with a 6×His tag (rVAR2), specifically binding pl-CSA, was expressed and purified from *E. coli* BL21 by inserting this fragment into a pET28a(+) plasmid that was then transferred into *E. coli*. BL21. A.** Screening of positive clones by PCR amplification (1-16, different clones amplified with PCR primers; M, DNA marker). **B.** Determination of the plasmid using restriction enzyme digestion (1 and 2, uncut plasmid; 3, plasmid digestion with BamHI; 4, plasmid digestion with BamHI and SalI; M, DNA marker). **C and D.** Determination of rVAR2 expression in the bacterial extract by reducing SDS-PAGE, followed by (C) Coomassie blue staining and (D) western blot analysis with total protein extract from (1-3) different positive clones and (4) the negative control. **E and F.** Determination of purification of rVAR2 using reduced SDS-PAGE, followed by Coomassie blue staining (E: 1, positive clone bacterial pellet; 2, bacterial culture supernatant following lysis and centrifugation; 3, unbound components; 4, bound components from the His tag column; 5, negative bacterial pellet; M, protein marker) and western blot analysis (F: 1, unbound components; 2, positive clone bacterial pellet; 3, bacterial culture supernatant following lysis and centrifugation; 4, bound components from His tag column; 5, negative bacterial pellet; M, protein marker) with total protein extract.

**Figure 2:**
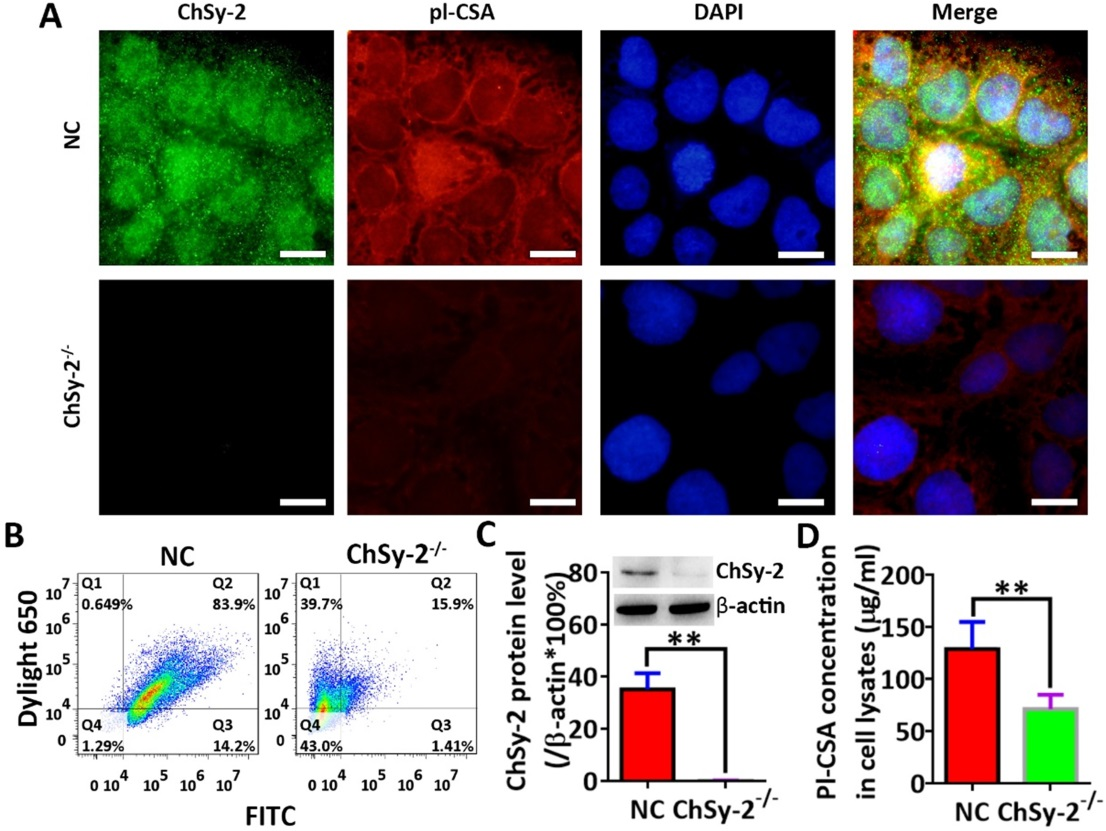
** The pl-CSA content was significantly downregulated through ChSy-2 knockout. A.** Immunofluorescence staining (green for ChSy-2, red for pl-CSA, blue for the nucleus, merged for color composition). Scale bar, 20 µm. **B.** Flow cytometry with FITC (the maximum absorption wavelength is 495 nm, and the maximum emission wavelength is 519 nm) and DyLight® 650 (the maximum absorption wavelength is 652 nm, and the maximum emission wavelength is 672 nm) representing ChSy-2 and pl-CSA, respectively. **C.** Western blot and analysis of integral optical density based on the protein band compared with β-actin. **D.** ELISA detection and analysis of pl-CSA content in choriocarcinoma. **P<0.01. pl-CSA, placental-like chondroitin sulfate A; ChSy-2, biosynthetic enzyme CS synthase-2.

Through prokaryotic expression and affinity purification, the electrophoretically pure rVAR2 was obtained for analysis of pl-CSA (Figure 1). The protein levels of ChSy-2 and contents of pl-CSA in the transfectants of positive cells and negative cells were determined by immunofluorescence, flow cytometry, western blots and ELISAs. Compared with that in the NC group, the green fluorescence density (ChSy-2) was below the threshold, and the red fluorescence density (pl-CSA) was significantly decreased in the ChSy-2^-/-^ group (Figure 2A and B). The ChSy-2 protein level was below the threshold (Figure 2C), and the pl-CSA content in the ChSy-2^-/-^ group was significantly decreased (Figure 2D) compared with that in the NC group. According to these data, ChSy-2, the biosynthetic enzyme of pl-CSA, was successfully eliminated in the ChSy-2^-/-^ group, and ChSy-2 knockout reduced the pl-CSA content in JEG3 cells.

### Reducing pl-CSA through ChSy-2 knockout impedes the functions of JEG3 cells *in vitro*

**Figure 3:**
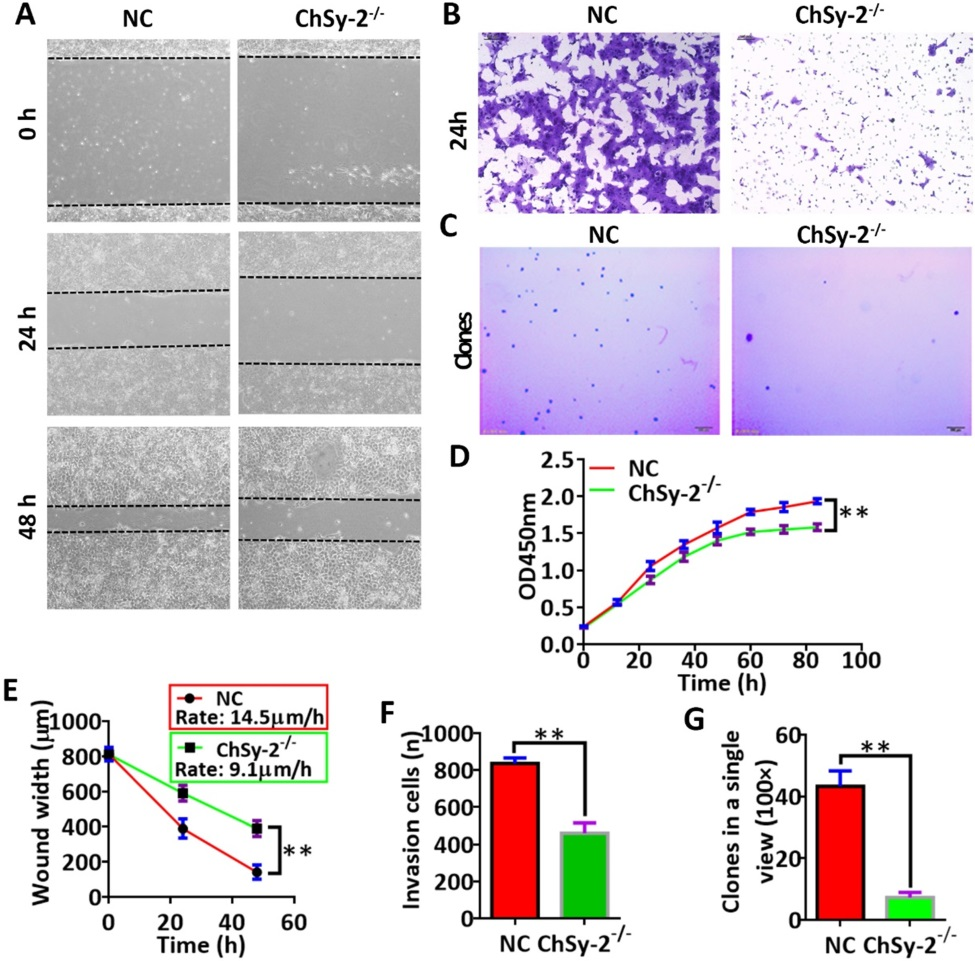
** Reducing pl-CSA through ChSy-2 knockout significantly impeded cell proliferation, migration, invasion and colony formation *in vitro*. A.** Scratch-wound assay for cell migration at 0, 24 and 48 h. Scale bar, 100 µm. **B.** Transwell assay for cell invasion at 24 h. Scale bar, 100 µm. **C.** Soft agar colony formation assay for colony formation *in vitro*. Scale bar, 100 µm. **D.** Cell proliferation index curve using Cell Counting Kit-8 assays. **E.** The analysis of the scratch-wound width and migration ratio (distance of cell migration at 1 h) of cells based on scratch-wound assays for 48 h. **F.** The analysis of invading cell number. **G.** Analysis of clone numbers in soft agar assays for tumorigenesis. **P<0.01. pl-CSA, placental-like chondroitin sulfate A; ChSy-2, biosynthetic enzyme CS synthase-2.

The proliferation, migration, invasion and colony formation capacities of the JEG3 cells were investigated by CCK-8, scratch-wound, transwell and soft agar colony assays, respectively. Compared with that in the NC group, the proliferation curve of the ChSy-2^-/-^ cells maintained a flat trend (Figure 3D), and the migration rate of the ChSy-2^-/-^ cells was significantly decreased (Figure 3A and E), and the number of invading cells was significantly lower (Figure 3B and F), and the number of clones was significantly lower (Figure 3C and G). According to these data, downregulation of ChSy-2/pl-CSA significantly impeded JEG3 cell proliferation, migration, invasion and colony formation *in vitro*.

### Reducing pl-CSA through ChSy-2 knockout inhibits tumorigenesis and metastasis of choriocarcinoma *in vivo*

**Figure 4:**
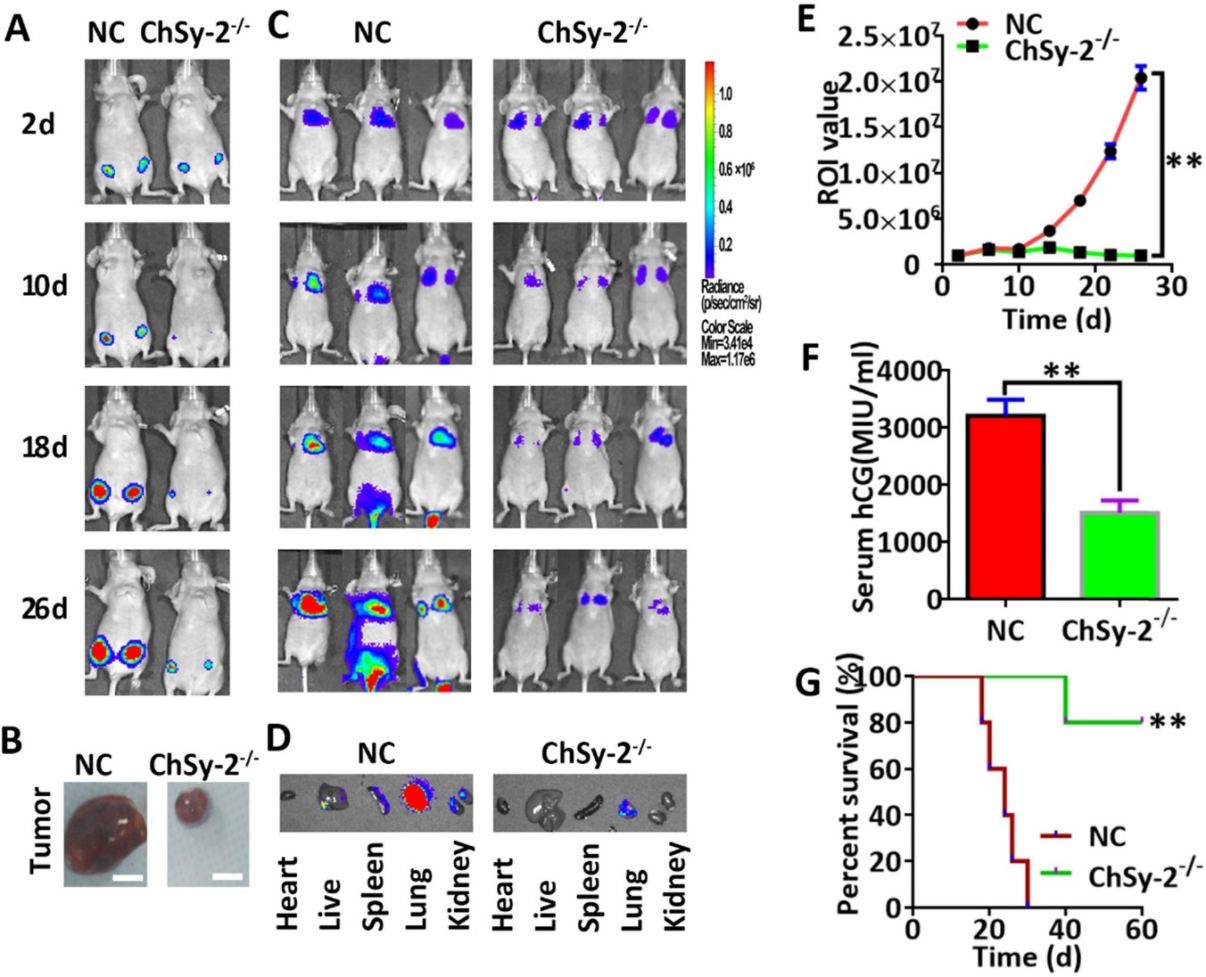
** Reducing pl-CSA through ChSy-2 knockout significantly inhibited tumorigenesis and metastasis of choriocarcinoma *in vivo*. A.** Monitoring of subcutaneous tumorigenesis through the IVIS imaging system with D-luciferin at 2, 10, 18 and 26 d *in vivo*. **B.** Volume detection of subcutaneous tumors. Scale bar, 0.5 cm. **C.** Monitoring of tumor metastasis after tail vein injection of modified JEG3 cell lines through the IVIS imaging system with D-luciferin at 2, 10, 18 and 26 d *in vivo*. **D.** The mouse models were euthanized, and the visceral organs, including the heart, liver, spleen, lung, and kidney, were observed for the fluorescence of metastatic tissues *ex vivo*. **E.** The region of interest (ROI) analysis of the whole body for tumor metastasis. **F.** Serum hCG levels. **G.** Percent survival of mice with subcutaneous tumorigenesis. **P<0.01. pl-CSA, placental-like chondroitin sulfate A; ChSy-2, biosynthetic enzyme CS synthase-2. hCG, human chorionic gonadotropin.

Following subcutaneous injection, the luciferase signals produced by the JEG3-Fluc2 cells were monitored in a tumorigenesis model. The tumor volume was continuously enlarged in the NC group over time. Interestingly, the tumor remained small in the ChSy-2^-/-^ group until the end of the experiment, and the percent survival in the ChSy-2^-/-^ group was significantly higher than that in the NC group (Figure 4A, B and G). Following tail vein injection, the luciferase signals produced by the JEG3-Fluc2 cells were monitored in a metastasis model. The luciferase signal was mainly located in the lungs for the first 10 days, with an insignificant difference, suggesting that the cancer cells were mainly in the lungs at the early stage in both the NC and ChSy-2^-/-^ groups. After 16 days, the luciferase signals in the NC group were significantly increased and transferred to the liver and kidney, suggesting that metastasis had occurred. Interestingly, the luciferase signals in the ChSy-2^-/-^ cells remained constant and even decreased (Figure 4C-E). Moreover, the serum hCG level in the ChSy-2^-/-^ mice was significantly lower than that in the NC mice (Figure 4F). Therefore, downregulation of ChSy-2/pl-CSA significantly inhibited tumorigenesis and metastasis of choriocarcinoma *in vivo*.

## Discussion

Choriocarcinoma has a high cure rate following chemotherapy with high-dose drug delivery, but it is associated with increased patient suffering 25. It remains a very promising approach to explore biotherapy targets. A previous study demonstrated that pl-CSA-targeted nanoparticles loaded with low-dose doxorubicin strongly inhibited the tumorigenesis and metastasis of choriocarcinoma *in vitro* and *in vivo* 26, suggesting that targeting pl-CSA could be a promising treatment for choriocarcinoma. However, the intrinsic function of pl-CSA in the tumorigenesis and metastasis of choriocarcinoma remains unclear. In this study, we further demonstrated that the pl-CSA was a promising biomarker for choriocarcinoma treatment, and the content of pl-CSA in tumor tissues might imply grade malignancy. Pl-CSA content could be efficiently reduced by targeting its biosynthetic enzyme ChSy-2, suggesting a promising approach to treat choriocarcinoma through inhibiting ChSy-2/pl-CSA pathway.

Glycosylation, a post-translational modification, exists in various biological processes, including physiological and pathological processes, in which the attachment of sulfated glycans to proteins plays an important role in biological functions and carcinogenesis 27. The conventional CSs, as sulfated GAGs, are assembled in Golgi compartments, and CS chain is covalently attached to its core proteins CSPGs, first through a tetrasaccharide linkage region linked to specific serine residues of the core protein and then by ligation with typical disaccharides 15. The tetrasaccharide linkage region is a general structure for a series of CS subtypes, as well as for some subtypes of heparin, dermatan and hyaluronic acid 15. The diverse CS chains are classified into CSA, CSB and CSC that depends on properties of disaccharide units by the corresponding specific glycosyltransferase and sulfotransferases, such as chondroitin synthase (ChSy, including ChSy-1, ChSy-2 and ChSy-3) and chondroitin polymerizing factor 15. Pl-CSA with specific and distinctive disaccharide units is specifically expressed in placental 28 and cancerous tissues 13. The structural properties of disaccharide units might facilitate pl-CSA interaction with placental or cancer bioactive molecules, thus for its function. However, it needs to further investigate the exact relationship between the pl-CSA and interacting molecules. Moreover, a presumed model of pl-CSA spatiotemporal expression might also happen in different stage of cancer development that might play a key role in tumorigenesis and metastasis of choriocarcinoma.

Targeting metabolic enzymes to study biopolysaccharides is an important technique, and metabolic enzymes were proposed as indirect targets for studying pl-CSA function. However, the enzymes involved in pl-CSA synthesis are unclear. Existing researches have confirmed that ChSy-2 has a higher glycosyltransferase activity for CSA than for other CSs and is also highly expressed in placental 17 and cancerous tissues 18; thus, it is involved in CSA production 29,30. According to the above thesis, we speculated that ChSy-2 may be the key synthase for pl-CSA production. In the present study, the pl-CSA content could be changed through regulating ChSy-2 expression, indicating that ChSy-2 is involved in pl-CSA biosynthesis, which may serve as a potential strategy for therapeutic intervention against glycan dysfunction. However, pl-CSA content could not be wiped out through knockout ChSy-2, suggesting that ChSy-2 was not the unique biosynthetic enzyme. In the process of CSPGs glycosylation, some other enzymes might be involved in pl-CSA biosynthesis, or partly compensated the ChSy-2 function. Meanwhile, this method has disadvantages; for example, the impact of ChSy-2 on tumorigenesis and metastasis prevents the full elucidation of glycan functions. Moreover, in the synthesis or metabolic process of pl-CSA, many enzymes were shown to have synergistic or sequential functions, suggesting that any changes related to any of the enzymes or the core proteins associated with pl-CSA metabolism would result in changes in other enzymes or core protein synthesis, according to the changes in pl-CSA content, to balance or compensate for the effect induced by the pl-CSA alterations. Therefore, the exact mechanism requires further investigation.

Pl-CSA, as a receptor for *P. falciparum*-infected red blood cells 28, is targeted by the VAR2CSA variant of *P. falciparum*-infected erythrocyte membrane protein 1 with a high affinity and specificity 31. VAR2CSA, as a transmembrane protein, includes 6 Duffy-binding-like domains (DBLs), in which the minimal binding region is ID1-DBL2Xb with a binding affinity of 21.8 nM 32. In the present study, a specific binding protein (rVAR2) was also recombined for pl-CSA detection. The signal of rVAR2, labeled with a 6×His tag, indicating specific binding with pl-CSA, was amplified through a DyLight® 650-labeled anti-6×His antibody that showed a high specificity, consistent with previous studies 33. This may provide an intervention or detection reagents for choriocarcinoma.

Pl-CSA, also as a pathological polysaccharide, may be an essential component for cell dissociation and invasion, cell-matrix interactions and clustering, as well as angiogenesis and metastasis in tumorigenesis 34. Over 90% of tumor tissues, including choriocarcinoma, breast tumor, melanomas, and so on, express pl-CSA on the cell surface and/or in the extracellular matrix (ECM) 14, 35. The capacities of cancer cells, including proliferation, adhesion, migration and invasion, are tightly associated with ECM, and the assembly and disassembly processes of ECM in cancer cells is required for tumorigenesis and metastasis cycling, suggesting spatiotemporal dynamically expression of specific ECM component of pl-CSA. Our results demonstrate that reduction of pl-CSA content by targeting ChSy-2 inhibited cell proliferation, migration and invasion *in vitro*, as well as inhibited tumorigenesis and metastasis of choriocarcinoma *in vivo*, which might be due to the change of the ECM assembly and disassembly processes, also be attributed to abnormal carbohydrate metabolism in tumor cells. Therefore, ChSy-2/pl-CSA system might be promising targets of cancer molecular therapy, but it requires further study.

In conclusion, pl-CSA, as a necessary component for JEG-3 cells, can be efficiently altered by targeting its biosynthetic enzyme ChSy-2. A reduction in pl-CSA content through ChSy-2 knockout inhibited choriocarcinoma tumorigenesis and metastasis through impeding tumor cells capacities of proliferation, migration and invasion. The present results indicated that glycometabolic dysfunction might be a key factor in tumorigenesis and metastasis, and ChSy-2/pl-CSA system might be promising biomarkers for the diagnosis and treatment of choriocarcinoma.

## Abbreviations

ChSy-2: chondroitin sulfate synthase 2
pl-CSA: placental-like chondroitin sulfate A
hCG: human chorionic gonadotropin
ELISAs: enzyme-linked immunosorbent assays
NC: negative control
